# Pasteurized Autograft-Prosthesis Composite Reconstruction May Not Be a Viable Primary Procedure for Large Skeletal Defects after Resection of Sarcoma

**DOI:** 10.1155/2017/9710964

**Published:** 2017-06-04

**Authors:** Seung Yong Lee, Dae-Geun Jeon, Wan Hyeong Cho, Won Seok Song, Chang-Bae Kong, Bum Suk Kim

**Affiliations:** Department of Orthopedic Surgery, Korea Cancer Center Hospital, Seoul, Republic of Korea

## Abstract

**Background:**

Among various types of composite biological reconstruction, pasteurized autograft-prosthesis composite (PPC) is popular when allograft is unavailable. Previous limited cohort study indicated result comparable to tumor prosthesis. However, as case number and follow-up increase, we experienced more complications than anticipated. We questioned the usefulness of PPC as a viable reconstructive option.

**Methods:**

We reviewed 142 PPCs and analyzed overall and location-related survival and factors associated with the failure of PPC.

**Results:**

Twenty-year survival rate of 142 PPCs was 39.8 ± 10.0%. Fifty-two (36.6%) of 142 PPCs showed failure. Among various locations, the proximal femur showed best survival: 78.0 ± 9.9%. Final status of the 52 failed PPCs was modular tumor prosthesis in 23 (43%), arthrodesis in 11 (21%), pseudarthrosis in 7 (13%), amputation in 7 (13%), and allograft-prosthesis composite in 4 (8%). Tumor volume > 200 cc (*p* = 0.001), pasteurization length ≤ 10 cm (*p* = 0.002), male sex (*p* = 0.02), and locations in pelvis or tibia (*p* = 0.029) were poor prognostic factors.

**Conclusions:**

Long-term survival of PPCs was below expectations. Despite the complexity of the procedure, there is little survival gain over tumor prosthesis. PPC may be indicated when a modular prosthesis is not readily available.

## 1. Introduction

The three major reconstructive options after resection of tumors involving major joints include use of a tumor prosthesis, an osteoarticular allograft, and a composite biological reconstruction [1–8]. Endoprosthetic reconstruction is the most popular method and has advantages such as immediate stability, short operative time, and relative suitability for patients who require adjuvant therapy. However, long-term mechanical complications and difficulty in soft tissue attachment are still problematic [9–11]. On the other hand, biological osteoarticular allografts have shown limited success [4, 12–14]. Composite biological reconstruction was developed to combine the advantage of a metallic prosthesis and biological method. Nevertheless, reports on composite reconstruction are sporadic and outcomes show wide variation [15–20].

Conceptually, composite biological reconstruction enables the restoration of bone stock, and the load-sharing properties of the allograft may contribute to the longevity of reconstruction. Among several types of composite biologic reconstruction, recycled autograft (pasteurized, irradiated, and frozen) is popular in situation when allograft is not readily available. We have used pasteurized autograft-prosthesis composite (PPC) as one of the reconstructive options since 1988 and have reported several small case series involving various locations.

Our previous limited study indicated that survival of PPC was comparable to that of tumor prosthesis in the femur, with a relatively high complication rate in pelvic locations and a lower rate of late infection in the tibia [18, 21–24]. However, as case numbers and follow-up increased, we experienced more complications than anticipated. Accordingly, we questioned whether PPC can substantiate its theoretical advantage in decreasing the failure rate. If the survival rate of PPC is comparable to or lower than that of a modular prosthesis, there is no reason to adopt this technically complex procedure.

This study of 142 PPC cases aimed to determine long-term survival and the incidence of complications, including nonunion, loosening, bony resorption, infection, and fracture, as well as the final reconstruction status of failed PPCs. A second aim was to identify the factors associated with PPC failure.

## 2. Materials and Methods

We retrospectively reviewed the records of 155 patients who underwent reconstructions using PPC between 1988 and 2014 at our institution. We excluded 13 patients because of (1) incomplete data (3 patients) or (2) less than 2 years of follow-up (10 patients). Therefore, the final study cohort comprised 142 patients. The indications for PPC were as follows: (1) predominance of osteoblastic pattern on plain radiography, (2) less than 1/3 cortical bone destruction on axial MRI, or (3) tumor confined to a single compartment. Data included patient's age, sex, pathologic diagnosis, initial tumor volume, location of tumor, length of pasteurization, fixation modality (cemented or noncemented), local recurrence, metastasis, and final survival status of patient (Table 1). The mean follow-up period was 110 months (range, 21–278). The follow-up duration was defined from the date of diagnosis to the date of death or the last visit. There were 90 males and 52 females, with an average age of 24 years (range: 4–72). Pathologic diagnoses included osteosarcoma in 112, chondrosarcoma in 15, Ewing's sarcoma in 5, malignant fibrous histiocytoma of bone in 2, metastatic carcinoma in 3, and others in 5. Average tumor volume was 186 cc (range: 6–2,167). The tumor volume was calculated from three parameters (length, width, and depth), using the ellipsoid formula: [*V* = (4*π*/3)*abc*]. Location of the tumor is as follows: 76 in the femur, 39 in the tibia, 12 in the humerus, and 15 in the pelvis. Average length of pasteurized bone was 14.7 cm (range: 5–35). Neoadjuvant and adjuvant chemotherapy was performed in 118 patients. There were 12 cases (8.5%) with local recurrence and 51 (36%) with distant metastasis. Patient final status was continuous disease-free in 86 (60.6%), no evidence of disease in 15 (10.6%), died of disease in 39 (27.4%), and alive with disease in 2 (1.4%).

Osteotomy was made at least 2 cm away from any evidence of tumor involvement, based on MRI. After resection of the tumor, the PPC was prepared as previously described. Briefly, (1) the bone was cleared of soft tissue and extraosseous tumor; (2) the medullary cavity was reamed and intraosseous tumor was removed; (3) the bone was then kept in preheated saline at 65°C for 30 minutes, retrieved, and prepared on a different table; and (4) after cylindrical reaming of bone, the assembled PPC was fixed to host bone. Implanted prostheses for composite reconstruction were as follows: femur (Link® Endo-Model® Modular Knee Prosthesis System, Germany; Zimmer VerSys® Hip System, USA), tibia (Link Endo-Model Modular Knee Prosthesis System), humerus (Depuy GLOBAL® Shoulder System, USA), and pelvis (Zimmer Trilogy® Acetabular System, USA).

We performed cemented fixation in 127 of 142 cases. Except for proximal tibial and pelvic locations (patients were immobilized in a plaster cast for 6 weeks), patients were allowed postoperative exercise with walking on crutches; unassisted, full weight-bearing was permitted at 4–6 months postoperatively.

Plain anteroposterior and lateral radiographic examinations were performed monthly until 2 years after the index operation. Radiographic union at the junctional site was assessed by one radiologist (Ji Young Yoo) and two of the authors (Dae-Geun Jeon, Seung Yong Lee). The site of the osteotomy was considered radiographically healed when callus was seen to be bridging the osteotomy line in both the anteroposterior and lateral planes. The radiographic interpretation of loosening followed the criteria of O'Neill and Harris [25]. Patients with radiologically demonstrated nonunion or loosening underwent no additional procedure unless they complained of symptoms in the affected limb. Prosthetic failure was defined as removal of the original prosthesis for any cause, or mechanical failure of the implant requiring original prosthesis removal. Time to failure was defined as the elapsed time between first surgery and date of prosthetic removal. After surgery, patients were seen every 3 months for the first 2 years and biannually thereafter. Functional results were assessed at final follow-up visits using the Musculoskeletal Tumor Society (MSTS) system [26]. Survival curves were determined using the Kaplan-Meier method and intergroup differences in survival were determined using the log-rank test. Multivariate analysis was performed using the Cox proportional hazards model. Analyses were performed using SPSS version 13.0 (SPSS Inc., Chicago, IL, USA), and* p* values of <0.05 were considered significant.

## 3. Results

**Figure 1:**
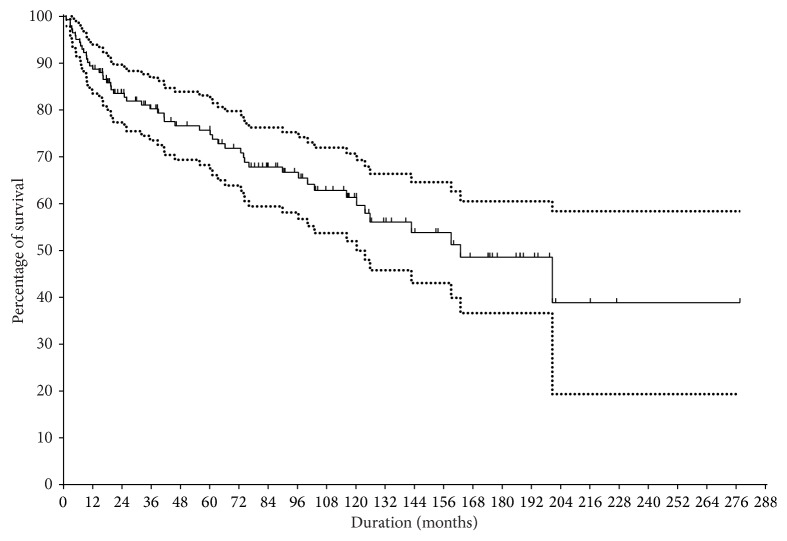
Survival of the implant, with removal of graft for any cause as the end point. Dotted line denote upper and lower 95% confidence interval.

**Figure 2:**
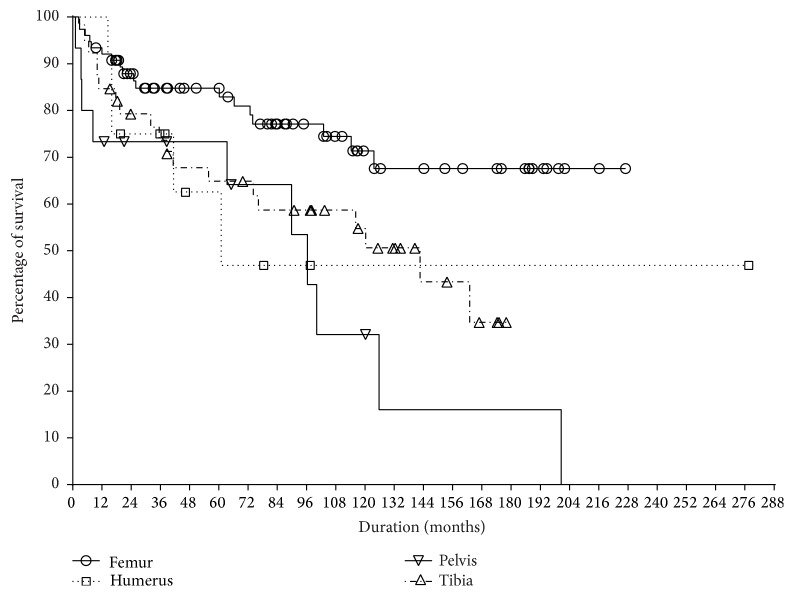
Survival of the implant according to the locations. Pelvic location showed the lowest survival (*p* = 0.006).

The 20-year survival rate of 142 PPCs was 39.8 ± 10.0% by Kaplan-Meier analysis (Figure 1). Fifty-two (36.6%) of 142 PPCs showed failure. The 20-year femoral survival rate was 67.6 ± 7.0%, the tibial rate was 34.7 ± 11.2%, the pelvic rate was 16.0 ± 13.5%, and the humeral rate was 46.9 ± 17.8 (Figure 2). The survival rate of distal femur was 65.8 ± 9.4%, while that of proximal femur was 78.0 ± 9.9%. In 90 patients who retained the prosthesis at the index operation, the average MSTS score was 23.5. The primary union rate of the osteotomy junction was 63% (90 of 142) and the average time to union was 22 months (6–132). Overall, the most frequent cause of failure was infection (35%), followed by loosening (23%), fracture of pasteurized bone (21%), metal failure (17%), and local recurrence (4%). The failure rate by location was femur in 24% (18 of 76), humerus in 42% (5 of 12), tibia in 49% (19 of 49), and pelvis in 67% (10 of 15). The main cause of PPC removal by location was infection in the femur (33%) and tibia (47%) and fracture of pasteurized bone in the pelvis (60%) and humerus (40%). Final limb status of the 52 failed PPCs was modular tumor prosthesis in 23 (43%), arthrodesis in 11 (21%), pseudarthrosis in 7 (13%), amputation in 7 (13%), and allograft-prosthesis composite in 4 (8%) (Table 2).

Resection length ≤ 10 cm (*p* = 0.002), locations in pelvis or tibia (*p* = 0.005), tumor volume > 200 cc (*p* = 0.001), male sex (*p* = 0.01), and noncemented fixation (*p* = 0.05) predicted worse survival on univariate analysis. Tumor volume > 200 cc (*p* = 0.001; relative risk [RR], 3.55; 95% confidence interval [CI], 1.90–6.63), pasteurization length ≤ 10 cm (*p* = 0.002; RR, 2.79; 95% CI, 1.47–5.29), male sex (*p* = 0.02; RR, 2.05; 95% CI, 1.05–4.00), and locations in pelvis or tibia (*p* = 0.029; RR, 1.95; 95% CI, 1.07–3.55) were independent poor prognostic factors for prosthesis survival on multivariate analysis (Table 3).

## 4. Discussion

In resections including major joints, use of a modular tumor prosthesis is generally regarded as a preferred reconstructive procedure; however, when tumor extent results in extensive loss of bone stock or when resection is performed in locations where soft tissue reconstruction is problematic, composite biological reconstruction can be considered. Because composite biological reconstruction simulates resurfacing arthroplasty, the theoretical complication rate of a composite will fall between that of conventional joint arthroplasty and tumor prosthesis. However, our previous small cohort PPC results for various locations did not show survival gain over tumor prosthesis [18, 21–24]. Furthermore, as case numbers and follow-up increased, we found that survival of PPCs deteriorated further. Therefore, we questioned the viability of this technically complex reconstruction as a viable primary procedure. The overall and by anatomical location long-term failure rates of PPCs were worse than those of tumor prostheses. Therefore, a PPC may be indicated when a modular prosthesis is not available, or in a proximal femoral location only.

This observational retrospective study is limited by a heterogeneous cohort, with use of different prosthesis designs and modes of fixation, various anatomical sites, use of chemotherapy, and amount of soft tissue resected. In addition, we did not compare the outcome of PPC use with that of our own tumor prosthesis cohort. We think that a comparison of our result with a published large cohort study on endoprosthetics would be more objective.

The PPC failure rate of 36.6% (52 of 142) and 20-year survival rate of 39% in the present study are consistent with those reported by Jeys et al. for endoprosthetic replacement [7]. One notable difference in our study is the higher rate of mechanical failure due to fracture of pasteurized bone or nonunion at the junctional site (Table 4). This may imply that PPCs are mechanically weaker than endoprostheses. Furthermore, a large-scale multicenter study on tumor prosthesis implantation by Henderson et al. showed a lower rate of failure than that using a composite biological reconstruction such as a PPC [27]. Pasteurized bone-prosthesis composite neither increased prosthesis survival nor decreased short- and long-term complications. At best, the results using PPCs are comparable to those for tumor prostheses. In this regard, questions arise on the rationale of performing this technically arduous procedure. The theoretical advantage of composite biological use is that it may increase the longevity of reconstruction by restoring host bone stock. However, two factors hinder such expectations. First, the high rate of delayed or nonunion at junctional sites increases the probability of stem loosening or stem fracture (especially in the distal femur). Second, the time-dependent resorption or fracture of pasteurized bone decreases the load-sharing property of recycled bone, thereby accelerating loosening or implant fracture. In this regard, junctional healing and resorption of graft are two important factors for the success of composite biologic reconstruction. Recycled autograft like pasteurized one is partially destroyed by tumor and its biologic and mechanical property deteriorates further after heat treatment. Therefore, compared to stout allograft, pasteurized autograft is weak and not suitable for composite biologic reconstruction.

Infection is the most common nonmechanical cause of failure. In our previous study, we confirmed a lower rate of late infection in the proximal tibia. In the present study, with a larger cohort and longer follow-up, an average time to infection of around 17 months after the index operation may suggest that composite biological reconstruction has a partially protective effect against late infection.

Salvage of a failed PPC is another concern. In most cases with mechanical failure, switching to a tumor prosthesis is a priority. However, several factors endanger sound revision. Loosening of a cemented stem usually accompanies marked osteoporosis or deformity of the diaphysis due to long-standing eccentric stem position. This makes it difficult to fix the new stem at the correct mechanical axis. Stem fracture usually occurs at a previous osteotomy site. Therefore, removal of a fractured intramedullary stem causes extensive damage of host bone, making subsequent rigid fixation of new stem problematic. As a result, revision of a failed PPC is more challenging than that of a tumor prosthesis. Although the main causes of PPC failure are perioperative infections and late mechanical complications, four clinical factors are also associated with failure. Large tumor volume seems to decrease mechanical strength of autografts, short pasteurization length is associated with nonunion (obtaining secure fixation at metadiaphyseal osteotomy junction seems to be difficult in PPC technique), pelvic or tibial location increases infection or fracture of autografts, and increased physical activity in males may lead to loosening.

In conclusion, long-term survival of PPCs was below expectations. Compared to that using a modular tumor prosthesis, this procedure neither decreased the complication rate nor increased the longevity of the prosthesis, and revision of a failed PPC is difficult. Considering the unsatisfactory long-term outcome and the complexity of the procedure, a PPC is recommended when a modular tumor prosthesis is not readily available.

## Figures and Tables

**Table 1:** Patients demographics (*n* = 142).

Characteristics	Number of patients (%)
Age	
≤14, >40	50 (35.2)
15–40 (*n* = 92)	92 (64.8)
Average 24 years (4–72)	
Gender	
Male	90 (63.3)
Female	52 (36.7)
Pathologic diagnosis	
Osteosarcoma	112 (78.9)
Chondrosarcoma	15 (10.6)
Ewing's sarcoma	5 (3.5)
Metastatic carcinoma	3 (2.1)
MFH, bone	2 (1.4)
Others^*∗*^	5 (3.5)
Initial tumor volume (cc)	
≤200 ml	100 (70.4)
>200 ml	42 (29.6)
Average	186.2
Location	
Femur	
Proximal	26 (18.3)
Distal	44 (30.9)
Total	6 (4.2)
Pelvis	15 (10.6)
Humerus	
Proximal	10 (7.1)
Total	2 (1.4)
Tibia	
Proximal	39 (27.5)
Pasteurization length (cm)	
≤10 cm	36 (25.4)
>10 cm	106 (74.6)
Fixation modality	
Cemented	127 (89.4)
Cementless	15 (10.6)
Local recurrence	
Proximal femur	5 (3.5)
Distal femur	3 (2.1)
Proximal tibia	4 (2.8)
Metastasis	51 (36)
Final status	
Continuous disease-free	86 (60.6)
No evidence of disease	15 (10.6)
Died of disease	39 (27.4)
Alive with disease	2 (1.4)
Follow-up	
Average 110 months (21–278)	

^*∗*^Each case of giant cell tumor, rhabdomyosarcoma, synovial sarcoma, hemangioendothelioma, and desmoplastic fibroma.

**Table 2:** Causes of 52 pasteurized bone-prosthesis composite failure and final status by location.

Tumor location (number of cases, percent)	Infection (percent)	LR	Fracture of pasteurized bone	Loosening + nonunion or resorption	Metal failure + nonunion	Final limb status				
						TP	Arthrodesis	Amputation	APC	Pseudo- arthrosis
Femur	6/18	0	3/18	5/18	4/18	8	3	6	1	0
(18/76, 24%)	(33%)	(0%)	(17%)	(28%)	(22%)					
Humerus	0	0	2/5	2/5	1/5	4	0	0	1	0
(5/12, 42%)	(0%)	(0%)	(40%)	(40%)	(20%)					
Pelvis	3/10	1/10	6/10	0	0	3^*∗*^	0	0		7
(10/15, 67%)	(30%)	(10%)	(60%)	(0%)	(0%)					
Tibia	9/19	1/19	0	5/19	4/19	8	8	1	2	0
(19/39, 49%)	(47%)	(5%)	(0%)	(26%)	(21%)					
Total	18/52	2/52	11/52	12/52	9/52	23	11	7	4	7
(52/142, 36.6%)	(35%)	(4%)	(21%)	(23%)	(17%)					
Average duration	16.8	47.7	87.7	68.1	60.4	56.4	36.8	24.0	94.9	53.8
(range)	(1.1–120.3)	(32.0–63.4)	(3.7–200.6)	(16.2–168.5)	(14.7–162.9)	(6.7–125.8)	(2.8–142.8)	(2.8–116.2)	(16.2–162.9)	(1.1–200.6)

LR: local recurrence, TP: tumor prosthesis, and APC: allograft prosthesis composite.  ^*∗*^Saddle prosthesis.

**Table 3:** Associations between clinical variables and pasteurized bone-prosthesis composite survival.

Variables	Univariate		Multivariate		
	20 y EFSR	*p* value	RR	95% CI	*p* value
Age					
≤14, >40 (*n* = 50)	56.9 ± 11.2	0.47	ND	ND	ND
15–40 (*n* = 92)	40.9 ± 9.4				
Gender					
Female (*n* = 52)	66.0 ± 8.9	0.01	1		
Male (*n* = 90)	31.1 ± 10.5		2.047	1.05–4.00	0.021
Initial tumor volume					
≤200 ml (*n* = 100)	40.7 ± 12.9	0.002	1		
>200 ml (*n* = 42)	44.3 ± 9.4		3.550	1.90–6.63	0.001
Location					
Femur, humerus (*n* = 88)	67.1 ± 6.3	0.003	1		
Pelvis, tibia (*n* = 54)	0		1.951	1.07–3.55	0.029
Pasteurization length					
>10 cm (*n* = 106)	52.5 ± 6.8	0.02	1		
≤10 cm (*n* = 36)	18.6 ± 14.3		2.785	1.47–5.29	0.002
Mode of fixation					
Cemented (*n* = 127)	37.0 ± 11.9	0.05	1		
Noncemented (*n* = 15)	33.3 ± 12.1		1.579	0.77–3.25	0.215
Use of chemotherapy					
Chemotherapy (*n* = 116)	38.2 ± 12.1	0.94	ND	ND	ND
Operation only (*n* = 26)	35.0 ± 16.5				
Total (*n* = 142)	42.6 ± 9.2				

10 y EFSR = 10-year event-free survival rate; RR = relative risk; CI = confidence interval; ND = not done.

**Table 4:** Comparison with previous studies.

Author	Case Nr/mean FU (years)	Type of prosthesis	Failure rate (%)	Implant survival (%)		Loosening (%)	Implant, pasteurized bone, periprosthetic fracture (%)	Infection (%)	LR (%)	Amputation (%)
				10-year	20-year					
Current Series	142/9.2	PPC	52/142 (36.6%)	59^*∗*^	39^*∗*^	8	14	12	1	9
Jeys et al.	661/10	TP	227/661 (34%)	58^†^	38^†^	11	4	11	5	10.6
Henderson et al.^‡^	2174/NA	TP	534/2174 (24.5%)	78	74	4	4	8	4	NA
Henderson et al.^§^	4359/NA	TP	1271/4359 (29.2%)	NA	NA	10	5.2	7.8	5.3	NA

Nr: number, FU: follow-up, PPC: pasteurized bone prosthesis composite, TP: tumor prosthesis, ^*∗*^implant removal for any cause, ^†^revision surgery for any cause, ^‡^result of five institutions, ^§^literature review in Henderson et al. study, and NA: not assessed.
